# Current and future distribution of *Forsythia suspensa* in China under climate change adopting the MaxEnt model

**DOI:** 10.3389/fpls.2024.1394799

**Published:** 2024-06-03

**Authors:** En Wang, Zongran Lu, Emelda Rosseleena Rohani, Jinmei Ou, Xiaohui Tong, Rongchun Han

**Affiliations:** ^1^ School of Pharmacy, Anhui University of Chinese Medicine, Hefei, China; ^2^ Institute of Systems Biology, Universiti Kebangsaan Malaysia, Bangi, Malaysia; ^3^ School of Life Sciences, Anhui University of Chinese Medicine, Hefei, China; ^4^ Anhui Province Key Laboratory of Research and Development of Chinese Medicine, Anhui University of Chinese Medicine, Hefei, China; ^5^ Joint Research Center for Chinese Herbal Medicine of Anhui of IHM, Anhui University of Chinese Medicine, Hefei, China

**Keywords:** *Forsythia suspensa* (Thunb.) Vahl, climate change, environment variables, habitat distribution, MaxEnt model

## Abstract

This study evaluated the potential impact of climate change on the distribution of *Forsythia suspensa*, a valuable traditional Chinese medicinal plant, using the MaxEnt model integrated with Geographic Information System (GIS). By analyzing occurrence data from various databases and environmental variables including climate and soil factors, we forecasted the present and future (2050s and 2070s) habitat suitability of *F. suspensa* under different greenhouse gas emission scenarios (RCP8.5, RCP4.5, RCP2.6). Results indicated that the suitable habitats for *F. suspensa* were primarily located in North, East, Central, Northwest, and Southwest China, with a significant potential expansion of suitable habitats anticipated by the 2070s, particularly under the high emission scenario. The study identified precipitation and temperature as the primary environmental drivers impacting the distribution of *F. suspensa*. Furthermore, a northward shift in the centroid of suitable habitats under future climate scenarios suggested a potential migration response to global warming. This work provides crucial insights into the future conservation and cultivation strategies for *F. suspensa* amidst changing climatic conditions.

## 1 Introduction

One of the significant ecological elements impacting the distribution and growth of plants is climate change (O'Connor et al., 2020). At the present time, population growth, rapid energy consumption, and increased carbon emissions are having an increasing impact on the global climate, not to mention the fact that world temperatures are going to increase continuously in the future (Tan et al., 2023). According to the study, the surface temperature on earth during 2011 to 2020 is 1.09°C higher compared with that in 1850–1906 (Ting et al., 2017). Relevant researches have shown that suitable habitats of plant migrate accompanying climate change, and future climate warming will cause vegetations to migrate to circumpolar latitudes and higher altitude regions. For example, *Stachys inflata* Benth was likely to migration higher elevations with consistent warming across Isfahan province (Shaban et al., 2023). And it also found that some temperature-sensitive plants in Finland migrate north (Habibullah et al., 2022).

Many models were developed to forecast the possible distribution of species in recent years including GARP (genetic algorithm for rule set prediction) (Adjemian et al., 2006), Enfa (ecological niche factor analysis) (Rosas et al., 2022), Bioclim (bioclimatic prediction system) (Booth et al., 2014), and the MaxEnt (maximum entropy approach) model (Mao et al., 2022). Now, the MaxEnt is considered the best tool to use in combination with the GIS (geographic information system) and has good accuracy. Studies showed that, compared with other models, MaxEnt model not only had a good prediction and stability (Duan et al., 2014), but also possessed the advantages of simple, easy to operate (Zhao et al., 2022) and small sample size. Therefore, it has become an ideal model for many scholars. Currently, this model is commonly used in the conservation of dying-out flora and fauna, the effect of climate variation on species, and the study of invasive species. For example, it was found the moderately suitable area of *Ligularia virgaurea* would expand significantly in northwest Sichuan, while the high-suitability area of *L. sagitta* would expand to eastern Tibet and western Sichuan in the 2050s and 2070s (Dong et al., 2023). In addition, Lee CM et al. found that the suitable area of fire ant *Solenopsis geminatawas* (Fabricius 1804) expanded and migrated to higher latitudes in the future by using MaxEnt (Lee et al., 2021).

*Forsythia suspensa* (Thunb.) Vahl, a shrubby plant, belongs to the genus *Forsythia* of Oleaceae. It is often found in China, Japan, Korea and many European countries. In China, it can be found in Sichuan, western Anhui, Shaanxi, Hebei, Shanxi, Henan, Hubei and Shandong provinces. The fruit of *F. suspensa*, a frequently prescribed traditional Chinese medicine, is divided “Qingqiao” and “Laoqiao” according to the collecting time (Pan et al., 2022). Green fruits that are beginning to ripen are gathered as Qingqiao, while fully ripened are gathered as Laoqiao (Wang et al., 2018). What is more, in the clinical treatment of Chinese medicine, *F. suspensa* is known as the “sacred medicine for sores” because of its remarkable curative effects. *F. suspensa* has many pharmacological effects and contains mainly forsythiaside, phillyrin, rutin, phillygenin and other active ingredients, which have anti-inflammatory, antibiosis and antiviral effects, etc. In addition, there is a difference in chemical component between Qingqiao and Laoqiao. It has been indicated that active ingredients, like forsythoside A, phillyrin, and rutin in Green Bridge were above Laoqiao (Bai et al., 2015). *F. Suspensa*, as one of bulk Chinese medicinal materials, is the raw material for many Chinese patent medicines, and the demand for it always exceeds the supply. Especially since the spread of the COVID-19, many proprietary Chinese medicines with obvious with pronounced heat-clearing efficacy contain *F. Suspensa*, which has led to a substantial increase in its demand.

At present, with the growing market demand, the resources of *F. suspensa* are becoming scarcer, which has become the key to the sustainable development of *F. suspensa*-related industries. And the standardized cultivation of *F. suspensa* is the key to its industrialization. Therefore, to understand the current regional distribution of *F. suspensa* in China and the trend of its fitness zone under future climate change, the present study was conducted using the ArcGIS software along with MaxEnt model to simulate and predict the prospective distribution area of *F. suspensa* within the present and future 2050s and 2070s under different greenhouse gas emission concentrations, which is quite important for protecting wild resources and standardized planting of for *F. suspensa.*

## 2 Materials and methods

### 2.1 Data collection

Adequate notes for the target plant are needed for the building of niche model. In this study, we collected a total of 302 specimens of the distribution records of *F. suspensa* from the Global Biodiversity Information Facility (GBIF, http://www.gbif.org) and Chinese virtual herbarium (CVH, http://www.cvh.ac.cn). For records without particular geographical coords, Baidu coordinate system is used to retrieve longitude and latitude by means of the geographical position described.

In this experiment, we used a total of 36 environment variables, including 19 climate parameters and 3 terrain factors obtained via the WorldClim (http://www.worldclim.Org) and 14 soil environmental elements from the Harmonized World Soil Database (HWSD, http://www.fao.org/faostat/en/#data) (**Supplementary Material 1**). These are now widely used to operate species distribution models and reflect the temperature and precipitation within the study area.

The representative concentration pathways (RCPs) can be represented by a range of integrated greenhouse gas (GHG) emission as well as concentration situations that can be regarded as input parameters for models projecting climatic variation due to the effect of behaviors of mankind in this century (Moss et al., 2010). It consists mainly of a mitigable options leading to a very low level of forcing (RCP2.6), two medium stabilization scenarios (RCP4.5/RCP6) and a very high baseline emission scenario (RCP8.5) (Vuuren et al., 2011). In this study, the potential future suitable area for *F. suspensa* was modeled under three typical concentration emission situations: RCP8.5, RCP4.5 and RCP2.6.

### 2.2 Data processing

#### 2.2.1 Analyzing and processing obtained occurrence data

**Figure 1:**
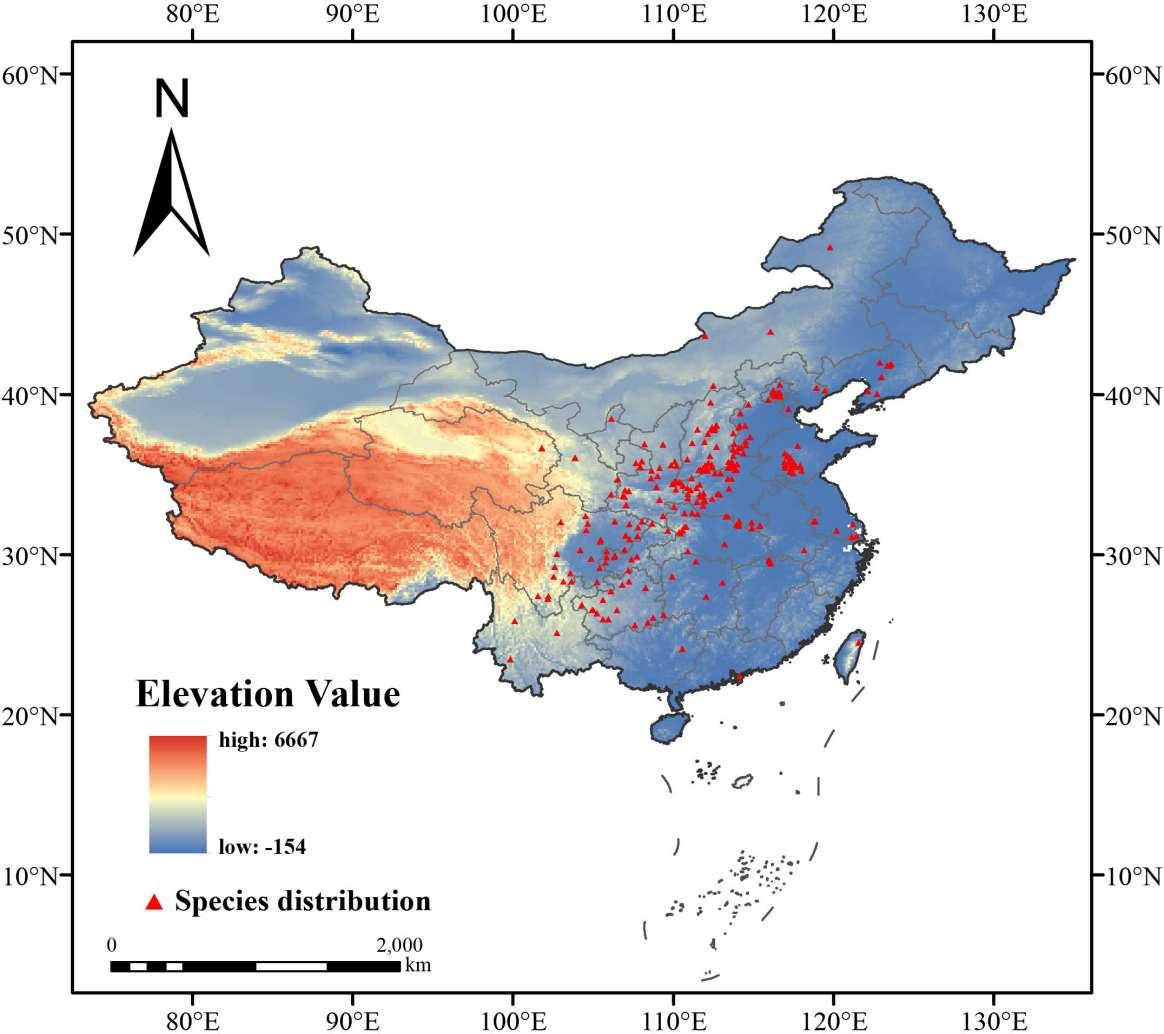
Distribution map of *F. suspensa* in China.

We examined the species distribution data and manually screened out incorrectly recorded and duplicate data. To avoid sampling errors generating localized distribution points and overfitting patterns, ArcGIS was utilized to ensure a maximum of one distribution point per 2.5 min grid by setting up buffers and cross-tabulation (Xu et al., 2023). As a result, we collected a total of 262 records of *F. suspensa* in China and mapped its detailed distribution (**Figure 1**).

#### 2.2.2 Analysis and processing of environmental variables

Spatial correlations could have an impact on the modeling process between many environmental variables. Therefore, environmental variables need to be screened. Firstly, 36 environmental data from 262 valid records were extracted using ArcGIS software, and then these data were input into SPSS software for Person correlation analysis to obtain the correlation coefficients. In this study, the one with low contribution rate among the two correlated variables was excluded, as the related environmental elements’ coefficient was bigger or equal to 0.8; in addition, for the size of the contribution rate of the environmental variables, we reserved the environmental factors with a contribution rate greater or equal to 0.5. Eventually, 11 bioclimatic elements were retained in all, like bio16 (precipitation of wettest quarter), bio12 (annual precipitation), bio6 (minimum temperature of coldest month), bio3 (isothermality), elev (elevation), slo (slope), t-sand (topsoil sand fraction), s_-_silt (bottom sediment content), t-clay (topsoil clay fraction), s-clay (bottom clay fraction) and t-gravel (top layer gravel volume percentage).

### 2.3 Model evaluation

AUC refers to the area under the ROC (receiver operating characteristic) curve which is usually utilized for testing the accuracy of a model, and it is not affected by the proportion of subjects in the analyzed sample (Parodi et al., 2022). AUC was frequently used in the evaluation of the performance of a variety of models for the Species Distribution Model (SDM) and is not impacted by the threshold setting. In this research, the magnitude of AUC values was utilized to assess the predictive effectiveness of respective models. Larger AUC values indicate greater correlation between modeled geographic distribution of target species and environmental elements, indicating that the predictive performance of this model is better (Ma et al., 2021). For the model, the prediction accuracy was categorized into five levels: excellent (0.9–1) (Zhao et al., 2021), good (0.8–0.9), fair (0.7–0.8), poor (0.6–0.7), and fail (0.5–0.6). And the feature combination (FC) and regularization multiplier (RM) are selected using the Akaike Information Criterion (AICc) to build the optimal model. In general, smaller AICc values suggest higher accuracy of the model’s predictions.

### 2.4 Changes of suitable habitat area and centroids

The final prediction results obtained after MaxEnt operation were imported into ArcGIS software and the reclassification tool was used to manually classify potential suitable areas of *F. suspensa*. The maximum test sensitivity plus specificity threshold (MTSPS) is used as the dividing line between suitable and unsuitable areas. Thus, the final classifications were high-suitability area (0.7–1.00), medium-suitability area (0.5–0.7), low-suitability area (0.3195–0.5) and non-suitable area (0–0.3195). The spatial extent of the four categories of areas was calculated and described.

To further study the change in the habitat of *F. suspensa* under current and different future scenarios, we used SDMtoolbox in ArcGIS toolkit to calculate the area of total suitable areas, highly suitable region, and the migration of centroids in different situations. Meanwhile, we also mapped and analyzed the changes in geographic distribution patterns, the migration paths of centroids and migration distances of normal region for *F. suspensa* under current and future diverse situations (Yan et al., 2021).

## 3 Results

### 3.1 Model variables as well as performance evaluation

**Figure 2:**
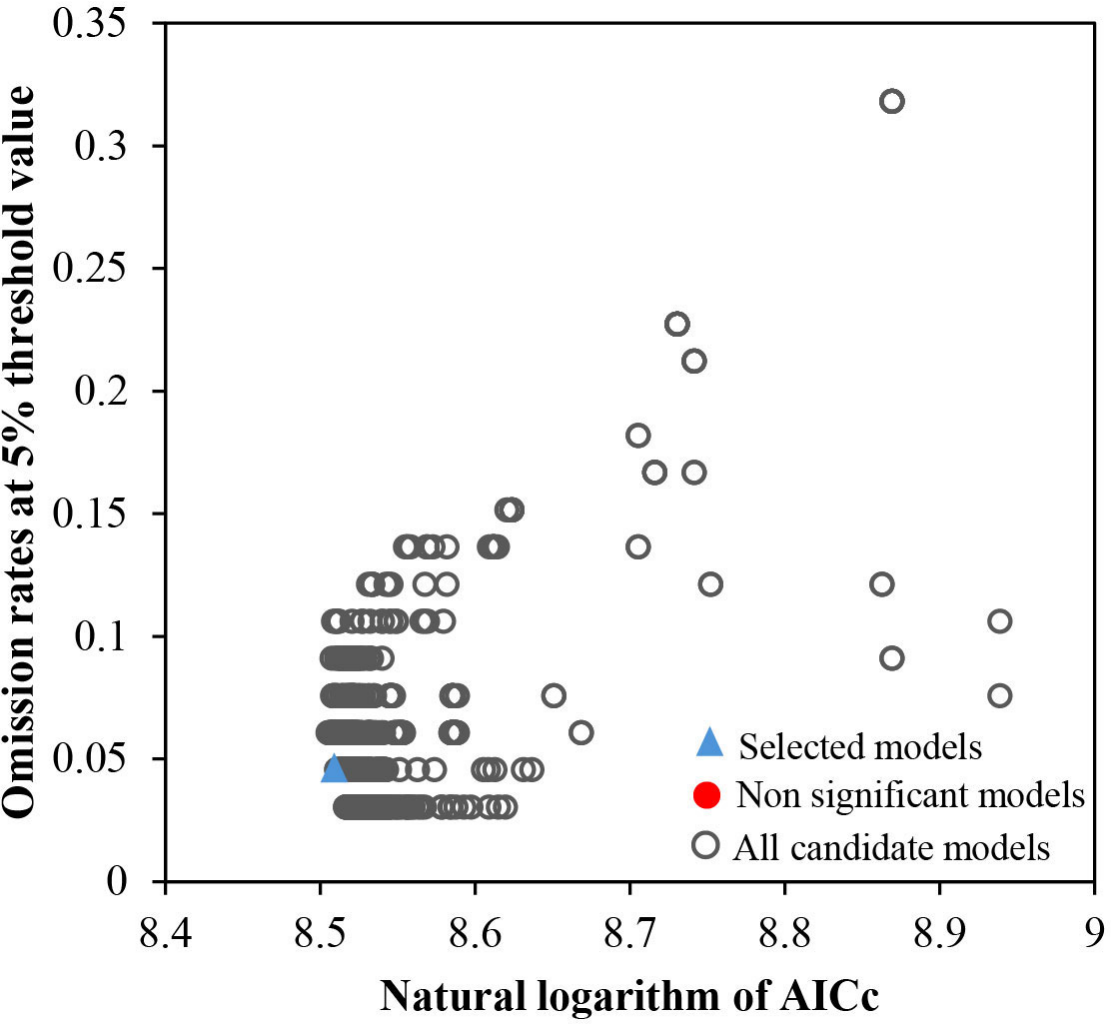
MaxEnt model parameter optimization results.

When using the MaxEnt to forecast the possible growth suitability zone of *F. suspensa* in diverse stages and contexts in China, we optimized the model to ensure accuracy and reliability of the results. By adopting the default settings (RM = 1, FC = lqpth), ΔAICc was 58.9989 with an omission rate of 0.06060. However, with the updated parameters (RM = 2, F= lqpt), ΔAICc was 0 with an omission rate of 0.04545, and the AUC value was as high as 0.898, which demonstrated the model was optimized to have favorable prediction accuracy and low overfitting (**Figure 2**; **Supplementary Material 2**). Therefore, compared with running the model with default parameters, optimizing the model first and then predicting the suitable region for *F. suspensa* under different climatic condition achieved higher accuracy.

### 3.2 Influence of major environmental factors

In this study, we analyzed the effect of each environmental element to the possible distribution area of *F. suspensa* using the MaxEnt model. Based on the results, it can be seen that bio6 (46.1%), bio16 (15.4%), and bio12 (14.4%) are the primary factors for model construction, and the accumulated contribution rate of the three factors is as high as 75.9%. The less influential environmental variables are elev (5.1%), bio3 (4.6%), s-clay (4.3%), t-gravel (3%), slope (2.5%), s-silt (2.3%), t-sand (1.4%), t-clay (0.9%), with a total contribution of 24.1% (**Table 1**).

**Table 1:** The contribution rate of environmental variables.

Variable	Percent contribution (%)	Permutation importance (%)	Suitable range
bio6	46.1	51	-33.5 – 13.6°C
bio16	15.4	11	344.4 – 625.1 mm
bio12	14.4	8.8	542.9 – 1408.8 mm
elev	5.1	13.9	-142.4 – 1039.3 m
bio3	4.6	0.8	15.5% – 31.46%
s_-_clay	4.3	2.6	5% wt., 7% wt., 10% wt., 16% wt., 21% wt., 30% wt., 33% wt., 44% wt., 51% wt.
t-gravel	3	2.3	2% vol., 5% vol., 6% vol., 16% vol., 19% vol.
slo	2.5	2.5	90.3 – 99.0°
s-silt	2.3	3.0	5% wt., 7% wt., 10% wt., 13% wt., 14% wt., 23% wt., 27% wt., 31% wt., 37% wt., 38% wt., 43% wt., 47% wt., 52% wt.
t-sand	1.4	2.6	19% wt., 22% wt., 28% wt., 29% wt., 35–38% wt., 47% wt., 53% wt., 76% wt., 78% wt., 81% wt., 83% wt., 89% wt., 90% wt.
t-clay	0.9	1.6	4% wt., 5% wt., 9% wt., 12% wt., 19% wt., 21% wt., 23–25% wt.,49% wt.

wt, weight; vol, volume.

**Figure 3:**
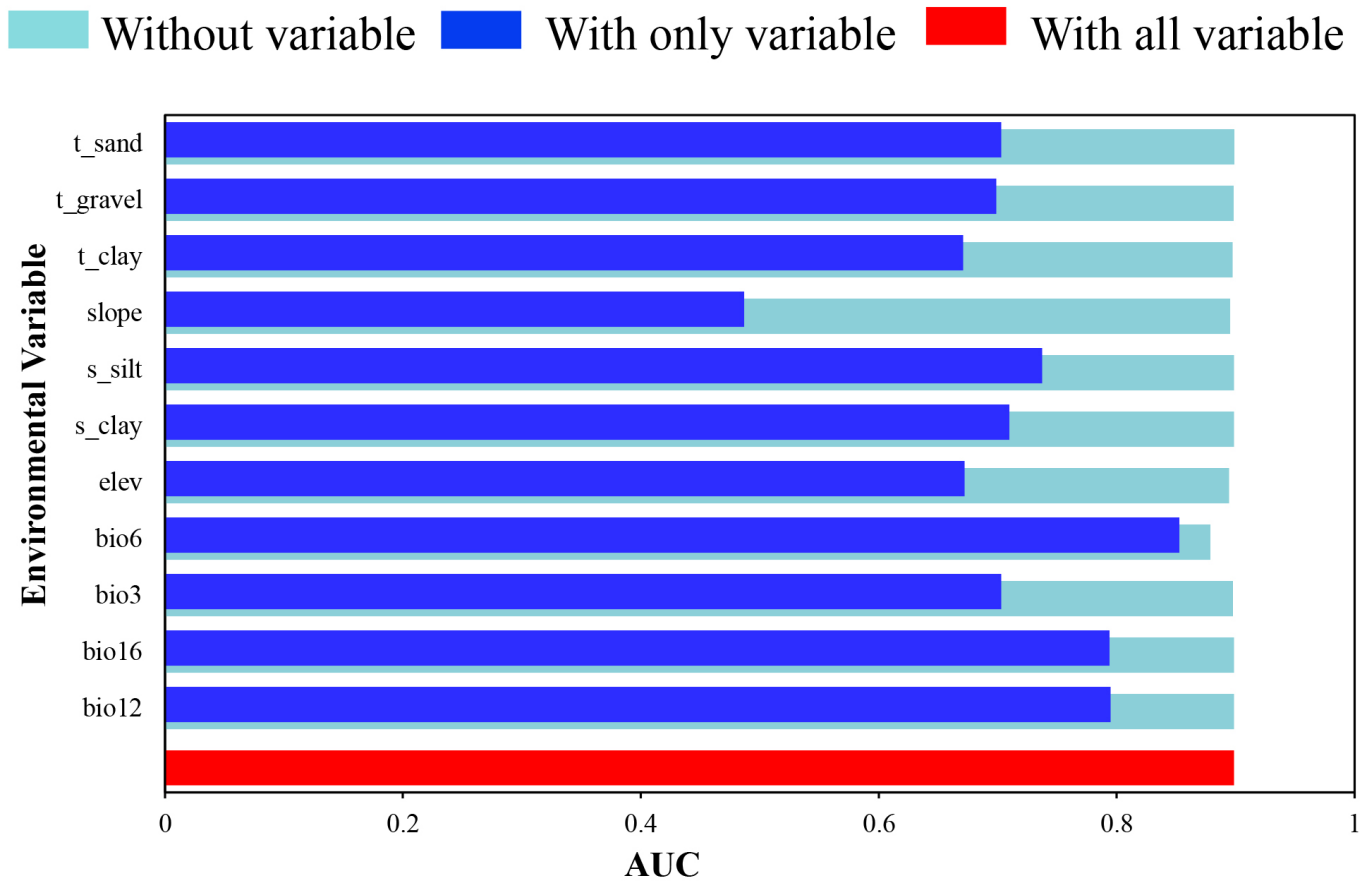
The results of the jackknife test of variable importance.

In addition, the significance of environmental factors on *F. suspensa* suspension was analyzed using the jackknife test. According to the outcome of the jackknife test, the AUC value of the model was greater than 0.85 when bio6 acted alone. This indicated that low temperature was the primary element affecting the distribution of suitable area of *F. suspensa*. Concurrently, the AUC values of bio12 and bio16 both fluctuated around 0.8, which also had a great influence on the prediction results. However, when slope was used alone, it did not have a great influence on the results (**Figure 3**; **Supplementary Material 3**). In summary, we concluded that bio6, bio16 and bio12 were the key environmental factors influencing the distribution of suitability regarding *F. suspensa*.

When the probability is greater than 0.5, the corresponding environmental factor value is conducive to plant growth. For example, according to bio6, the distribution probability of *F. suspensa* increases starting from -33.5°C and reaches its maximum value (0.52) at -14.3°C, and then decreases back to 0.5 at 13.6°C. Therefore, the suitable range of bio6 for *F. suspensa* growth is -33.5 – 13.6°C. Concerning bio16, within the range of precipitation from 344.4 mm to 625.1 mm, the maximum probability (0.60) of *F. suspensa* distribution is achieved at 387.6 mm (**Table 1**; **Supplementary Material 4**).

### 3.3 Current potential distribution of *F. suspensa* in China

**Figure 4:**
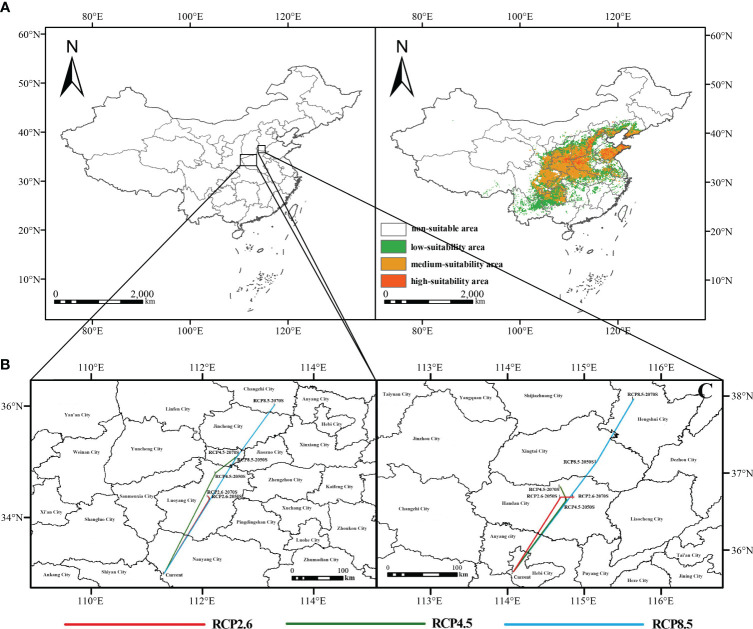
Present distribution of potential habitat and mass center migration. **(A)** present potential distribution region; **(B, C)** mass center migration concerning the three climate situations.

Under the present weather, the potential habitat of *F. suspensa* is mainly located in North, East, Central, Northwest and Southwest China. In addition, the total area of the normal area of *F. suspensa* reaches 1.7154 × 10^6^ km^2^, approximately 17.87% of China’s total land area. The area of the low suitability is 7.557× 10^5^ km^2^, which accounts for approximately 7.87% of the total surface level. It is distributed in Yunnan, southern Guizhou, Hubei, Hunan, Jiangxi, Zhejiang, Anhui provinces and other areas. The area of the moderate suitability zone is 8.623 × 10^5^ km^2^, approximately 8.98% of the total surface level. It is mainly distributed in the eastern part of Sichuan, Henan, Shaanxi, most of Gansu and Ningxia bordering Shaanxi provinces. The total area of high-suitability area is 9.74×10^5^ km^2^, approximately 1.01% of the total land area of the country, and is primarily situated in Shandong, Shaanxi, Henan, Hebei, and Shanxi provinces (**Figure 4A**; **Supplementary Material 5**).

### 3.4 Predicted potential distribution of *F. suspensa* in China in the future

**Figure 5:**
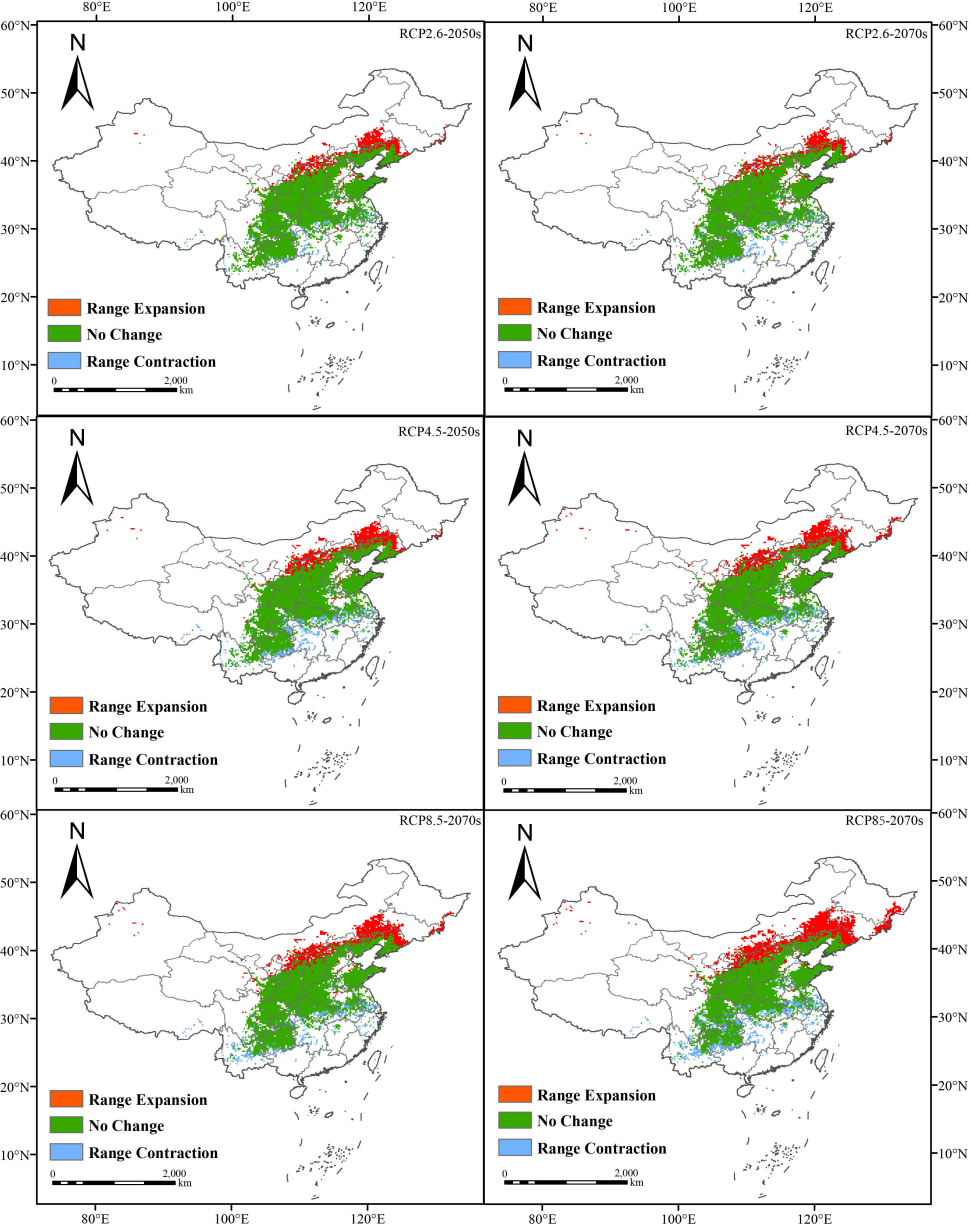
Change on geographical and spatial pattern concerning the overall suitable area in 2050s and 2070s in contrast with the present (blue: contraction area, green: stability area, red: expansion area).

We forecasted the distribution of normal areas for *F. suspensa* in China under six different climatic conditions. The outcome indicated that its total normal growth area would increase under the effect of climatic variation in the future. And the acreage of the proper area would be as large as 1.9514 × 10^6^ km^2^ under the RCP8.5 GHG emission concentration in the 2070s (**Figure 5**; **Supplementary Materials 6**, **7**).

Moreover, changes in highly suitable areas were more relative to changes in the total area of proper areas. For different GHG emission scenarios including RCP2.6, RCP4.5 and RCP8.5, with the increased concentration of greenhouse gases, the size of the high-suitability zone showed a decreasing trend in the 2050s. It decreased from 1.07 × 10^5^ km^2^ to 1.023 × 10^5^ km^2^ and then to 9.91 × 10^4^ km^2^, which accounted for 1.11%, 1.07% and 1.03% of the total land area of the country. However, unlike in the 2050s, the size of the high-suitability area in the 2070s fluctuated despite higher GHG concentrations, first from 1.039 × 10^5^ km^2^ to 1.054 × 10^5^ km^2^, and then decreased to 1.029 × 10^5^ km^2^, which accounted for 1.08%, 1.10%, and 1.07% of the total area.

**Figure 6:**
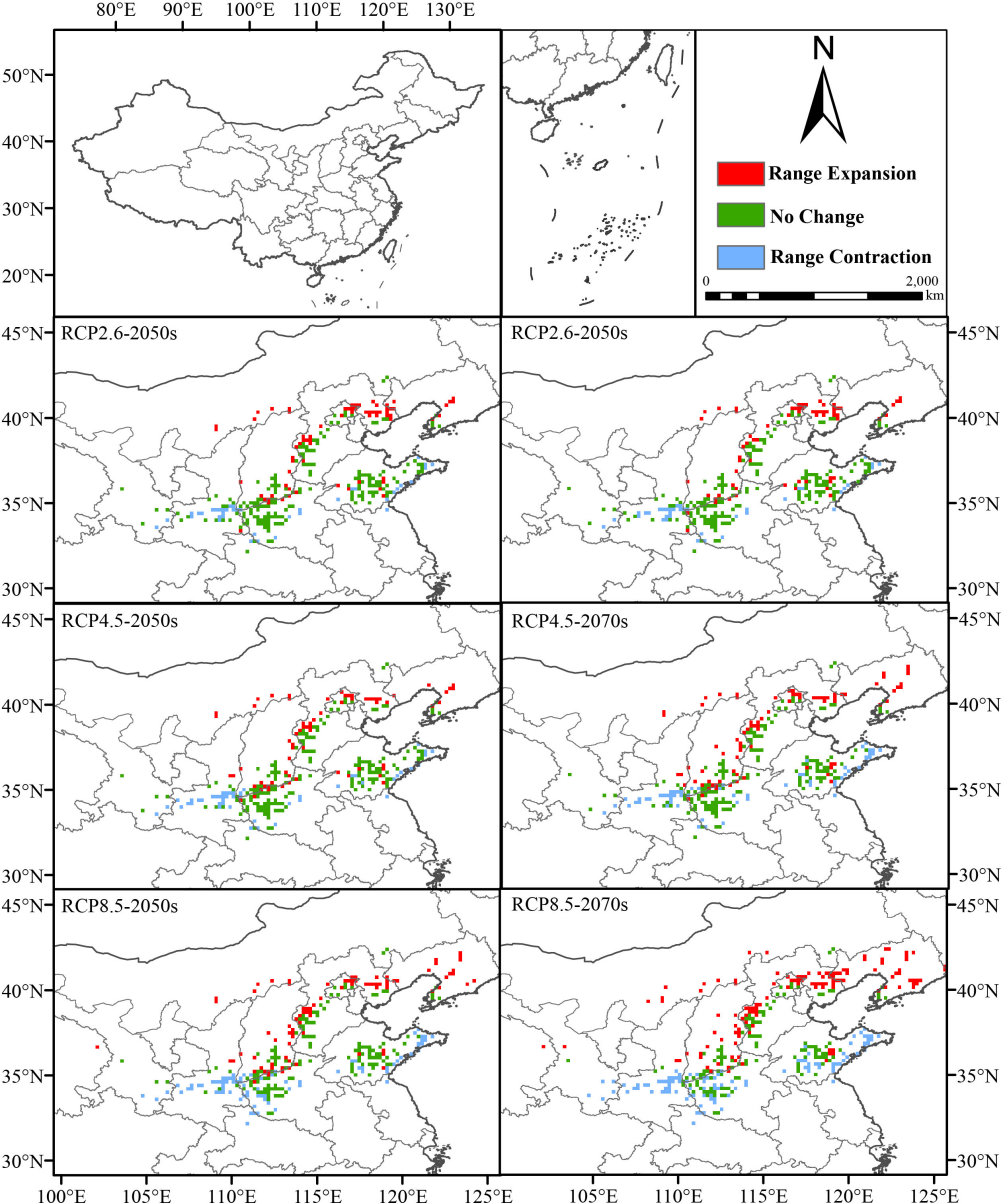
Change on geographical and spatial pattern concerning the high-suitability area for *F. suspensa* in the 2050s and 2070s in contrast with the present (blue: contraction area, green: stability area, red: expansion area).

We analyzed the changes in the high-suitability area of *F. suspensa* over time when the GHG emission concentrations were the samec (RCP2.6 scenario), the size of the high-suitability area increased with time from the current 9.74 × 10^4^ km^2^ to 1.07 × 10^5^ km^2^, and then decreased to 1.039× 10^5^ km^2^. Concerning the RCP4.5 situation, the total square measure of highly suitable region increased over time, first to 1.023× 10^5^km^2^ and then continued to increase to 1.054 × 10^5^ km^2^. Regarding the RCP8.5 situation, the overall area of high-suitability region mounted from the current time to 9.91 × 10^4^ km^2^ and then to 1.029 × 10^5^ km^2^. Overall, the size of the potential high-suitability area for *F. suspensa* is closely related to the concentration of GHG in the tested future periods (**Figure 6**; **Supplementary Material 8**).

### 3.5 Centroids migration of suitable areas in the coming period

Based on the output of the model, we used SDMtoolbox in ArcGIS v10.4 to analyze the centroid movement of *F. suspensa* over time under different GHG emission situation and plotted the trajectory of center-of-mass change (Wu et al., 2022). When we take the MTSPS value (MTSPS = 0.3159) as the threshold, the current center of mass was situated in Xichuan County, Henan Province (111.314°E, 32.9972°N). From now to the 2050s, centers of mass in the three situations of RCP8.5, RCP4.5 and RCP2.6 moved by 16.68 km, 21.75 km, and 25.73 km at different angles. And the center of mass is situated in Yiyang County, Luoyang City (112.130°E, 34.3364°N), Xin’an County, Luoyang City (112.236°E, 34.7996°N) and Jiyuan City, Henan Province (112.595°E, 35.0557°N), respectively. From the 2050s to the 2070s, the center of mass shifted by 0.62 km, 5.15 km, and 12.51 km under the RCP8.5, RCP4.5, and RCP2.6 scenarios. The centroids were situated in Yiyang County, Jiyuan City and Huguan County, respectively (**Figure 4B**). In a word, under different GHG emission situation, the possible normal area of *F. suspensa* will go north.

Changes in the highly suitable zone when the threshold was set at 0.7 were also assessed and the current center of mass was situated in Qi County, Henan Province (114.078°E, 35.7178°N). However, from the present to the 2050s, the center of mass in the three situations RCP8.5, RCP4.5 and RCP2.6 shifted to the northwest by 12.09 km, 12.11 km and 18.55 km at different angles. The shifted centers of mass were situated at 114.691°E/35.7178°N, 114.77°E/36.654°N and 115.159°E/37.1433°N, respectively. From the 2050s to the 2070s, the center of mass shifted by 1.55 km, 1.99 km and 10.05 km for the three emission scenarios, respectively. The shifted centers of mass were situated in Quzhou County (114.866°E, 36.6875°N), Handan City (114.687°E, 8203°N) and Shenzhou City (115.645°E, 37.9622°N) (**Figure 4C**). Based on our analysis, under different GHG emission concentrations, there will be a northward migration of high-suitability area for *F. suspensa* in the future.

## 4 Discussion

### 4.1 MaxEnt model’s predictive ability

At present, the MaxEnt model is widely used to study normal region of various plants under climate change (Xia et al., 2023). Based on the information collected from *F. suspensa* sample sites, we utilized the MaxEnt model to forecast the possible normal region of this plant in China. The simulation showed the suitable region of *F. suspensa* in China were principally distributed in Yunnan, Guizhou, Sichuan, Hubei, Hunan, Shanxi, Henan, Jiangxi and Hebei provinces. And we optimized the model with ΔAICc = 0 and AUC > 0.8, which indicated that the MaxEnt model was calculable and accurate in forecasting the distribution of *F. suspensa.*

### 4.2 The impact of environmental factors on distribution of *F. suspensa*

Studies have shown that rainfall and temperature are the primary elements affecting plant growth and reproduction (Dong et al., 2023). We identified the key environmental elements influencing changes in suitable areas for *F. suspensa* through jackknife test and model-calculated contributions of climatic variables and the results indicated that bio6, bio16 and bio12 were the most important elements. As a shrub, *F. suspensa* prefers to grow in warm, humid climates, so rainfall and temperature can have a huge influence on its growth. Water is crucial for the survival of plants, as its shortcomings limit their growth and development, ultimately affecting yield (Leisner et al., 2023). Sufficient precipitation will increase the water content in the soil, which is favorable to the growth and reproduction of *F. suspensa*. However, drought causes *F. suspensa* seedling shortage, fruit growth retardation and fruit wilting, seriously affecting the yield and quality. Although it needs more water during the nutritive growth period, too much precipitation is not conducive to the normal growth of *F. suspensa*. Excessive moisture in the soil may be detrimental to plant growth and development by causing hypoxia, unhealthy root growth and increased energy expenditure (Pais et al., 2022). For example, the response of photosynthesis to soil moisture in *F. suspensa* through potting experiments in a controlled greenhouse was trialed. It was found that the ability of the plant to carry out photosynthesis was related to soil moisture in spring and summer seasons. It has also been shown that in areas with very high soil moisture content, *F. suspensa* can suffer from undesirable conditions such as prolonged branching, fewer blooms, and low fruiting (Lang and Wang, 2015).

Additionally, temperature is other pivotal factor that affects the normal growth of *F. suspensa*. When the ambient temperature is below the lowest temp for plant growth, it is easy to generate frost damage to the plant. For example, low temperature in spring will cause peroxidation of cell membrane lipids in *F. suspensa* leaves and increase cell permeability, resulting in accumulation of MDA (Li et al., 2023). In addition, plant flowering is closely related to temperature. If *F. suspensa* flowering encounters low temperature conditions, it can damage the flower buds, thus affecting the yield (An, 2009). This study showed that rainfall and temperature affect the possible geographic distribution of *F. suspensa* in China and therefore as far as its future distribution is concerned, such parameters will also exert considerable impact.

### 4.3 Changes in spatial pattern and centroid

According to our findings, from now until the 2070s, the total acreage of normal region showed a sustained expansion trend. Especially in the 2070s, the total acreage of normal area increased under different concentrations of the three greenhouse gas emissions. In addition, the centroid of the plant’s normal region shifted under climatic variation. It was found that the mass center of the total suitable region of *F. suspensa* was situated in Xichuan County, Nanyang City, Henan Province, and the center of the highly suitable region was in Qi County, Hebi City, Henan Province. The centroid for different periods and emission situations was situated in the north of the present mass center. In other words, the center moved to higher latitudes. Based on the predicted findings, with the impact of future climate change, variation of space position of suitable region for *F. suspensa* basically coincided with the movement of the centroid, with northward expansions. This finding is in line with relevant research discoveries studying the impact of global warming that some species will migrate to higher latitudes or altitudes (Gu et al., 2021). For example, *Castanopsis hystrix* Miq. tends to expand to the northeast area at high latitudes under the ssp5–8.5 climate scenario (Shen et al., 2023). Therefore, when planting *F. suspensa*, it is needful to consider the influence of future climatic change and primary environmental elements on its suitability zones.

### 4.4 Study limitations

The shortcoming of this research lies firstly in the sample information because we used the present distribution data of *F. suspensa* to forecast its future possible normal region, and the results are theoretical speculations. Secondly, the selected 36 environmental factors cannot fully represent all elements impacting the geographical distribution of *F. suspensa*. Other elements like illumination, air, species interactions and anthropogenic impacts on species distribution need to be considered (Gu et al., 2021). Therefore, the impacts of other elements on species distribution modeling need to be considered and further investigated.

## 5 Conclusion

Our work investigated the influence of climate change on the distribution of *F. suspensa*, a significant traditional Chinese medicinal plant, utilizing the MaxEnt model to predict its current and future habitat suitability under various climate scenarios. We collected extensive occurrence and environmental data, optimizing the MaxEnt model to accurately forecast *F. suspensa*’s distribution across China, emphasizing the importance of temperature and precipitation as key environmental factors influencing its growth.

Under current conditions, *F. suspensa* predominantly occupies North, East, Central, Northwest, and Southwest China, covering approximately 17.87% of China’s land area. Predictions for future scenarios suggested an expansion of suitable habitats, especially under the RCP8.5 scenario by the 2070s, despite a decrease in the proportion of highly suitable areas in the 2050s. This expansion aligned with the northward shift of the plant’s habitat centroid, indicating a response to global warming by migrating to higher latitudes.

The MaxEnt model’s reliability in predicting the distribution of *F. suspensa* and its suitability regions under climate change was assessed. We announced the critical role of rainfall and temperature in determining the plant’s geographical distribution and potential shifts due to climate change. The study anticipated a northward migration of *F. suspensa*’s suitable habitats in response to future climatic variations, consistent with broader observations of species adaptation to global warming.

This research contributes to understanding the effects of climate change on medicinal plants and assists in strategic conservation and cultivation planning to ensure the sustainable supply of *F. suspensa*, highlighting the intersection of traditional medicine, ecology, and climate science.

## Data availability statement

The original contributions presented in the study are included in the article/**Supplementary Material**. Further inquiries can be directed to the corresponding authors.

## Author contributions

EW: Data curation, Formal analysis, Investigation, Writing – original draft. ZL: Formal analysis, Methodology, Software, Writing – original draft. ER: Visualization, Writing – original draft. JO: Resources, Validation, Writing – original draft. XT: Project administration, Supervision, Writing – review & editing. RH: Conceptualization, Supervision, Writing – review & editing, Funding acquisition.
