# Supplementary material for: Current and future distribution of Forsythia suspensa in China under climate change adopting the MaxEnt model
Source: Front Plant Sci. 2024 Jun 3;15:1394799. doi: 10.3389/fpls.2024.1394799 (PMC11180877; doi:10.3389/fpls.2024.1394799)
Supplement: Supplementary file 1 [file DataSheet_1.zip › Supplementary Material/Supplementary material 1.docx]

Supplementary material 1. Environment variables

| **Factor type** | **Code** | **Environment factor** | **Unit** | **Factor after decorrelation** |
| --- | --- | --- | --- | --- |
| Climate factor | BIO1 | Annual mean temperature | ℃ | Eliminated |
|  | BIO2 | Mean diurnal range | ℃ | Eliminated |
|  | BIO3 | Isothermality | % | Selected |
|  | BIO4 | Temperature seasonality | % | Eliminated |
|  | BIO5 | Max temperature of warmest month | ℃ | Eliminated |
|  | BIO6 | Min temperature of coldest month | ℃ | Selected |
|  | BIO7 | Temperature annual range | ℃ | Eliminated |
|  | BIO8 | Mean temperature of wettest quarter | ℃ | Eliminated |
|  | BIO9 | Mean temperature of driest quarter | ℃ | Eliminated |
|  | BIO10 | Mean temperature of warmest quarter | ℃ | Eliminated |
|  | BIO11 | Mean temperature of coldest quarter | ℃ | Eliminated |
|  | BIO12 | Annual precipitation | mm | Selected |
|  | BIO13 | Precipitation of wettest month | mm | Eliminated |
|  | BIO14 | Precipitation of driest month | mm | Eliminated |
|  | BIO15 | Precipitation seasonality | % | Eliminated |
|  | BIO16 | Precipitation of wettest quarter | mm | Selected |
|  | BIO17 | Precipitation of driest quarter | mm | Eliminated |
|  | BIO18 | Precipitation of warmest quarter | mm | Eliminated |
|  | BIO19 | Precipitation of coldest quarter | mm | Eliminated |
| Soil factor | T_SAND | Topsoil sand fraction | %wt. | Selected |
|  | S_SAND | Bottom sand fraction | %wt. | Eliminated |
|  | T_CLAY | Topsoil clay fraction | %wt. | Selected |
|  | S_CLAY | Bottom clay fraction | %wt. | Selected |
|  | T_SILT | Surface silt particle content | %wt. | Eliminated |
|  | S_SILT | Bottom sediment content | %wt. | Selected |
|  | T_GRAVEL | Top layer gravel volume percentage | %vol. | Selected |
|  | S_GRAVEL | Volume percentage of bottom gravel | %vol. | Eliminated |
|  | T_PH_H2O | The top lay acidity and alkalinity | -log(H^+^) | Eliminated |
|  | S_PH_H2O | The bottom layer acidity and alkalinity | -log (H^+^) | Eliminated |
|  | T_ESP | Top exchangeable sodium salt | % | Eliminated |
|  | S_ESP | Bottom exchangeable sodium salt | % | Eliminated |
|  | T_BULK_DEN | Topsoil bulk density | kg/dm^3^ | Eliminated |
|  | S_BULK_DEN | Soil bulk density | kg/dm^3^ | Eliminated |
| Terrain factor | SLO | Slope | 。 | Selected |
|  | ASP | Aspect | 。 | Eliminated |
|  | ELEV | Elevation | m | Selected |
