# Supplementary material for: Current and future distribution of Forsythia suspensa in China under climate change adopting the MaxEnt model
Source: Front Plant Sci. 2024 Jun 3;15:1394799. doi: 10.3389/fpls.2024.1394799 (PMC11180877; doi:10.3389/fpls.2024.1394799)
Supplement: Supplementary file 1 [file DataSheet_1.zip › Supplementary Material/Supplementary material 5.docx]

Supplementary material 5. The sum of the total suitable area of *F. suspensa* in current situation

| **the suitable area** | **The total area(km^2^)** | **Percentage of total land area in China (%)** |
| --- | --- | --- |
| Total suitable area | 1.7154 × 10^6^ | 17.87 |
| Low-suitability area | 7.557 × 10^5^ | 7.87 |
| Medium-suitability area | 8.623 × 10^5^ | 8.98 |
| High-suitability area | 9.74 × 10^5^ | 1.01 |
