# Supplementary material for: Current and future distribution of Forsythia suspensa in China under climate change adopting the MaxEnt model
Source: Front Plant Sci. 2024 Jun 3;15:1394799. doi: 10.3389/fpls.2024.1394799 (PMC11180877; doi:10.3389/fpls.2024.1394799)
Supplement: Supplementary file 1 [file DataSheet_1.zip › Supplementary Material/Supplementary material 6.docx]

Supplementary material 6. The sum of the total suitable area of *F. suspensa* in the future scenario

| **GHG emission scenarios** | **Total suitable area of total area(km^2^)** | **Low-suitability area of total area(km^2^)** | **Medium-suitability area of total area(km^2^)** | **High-suitability area of total area(km^2^)** |
| --- | --- | --- | --- | --- |
| 2050s RCP2.6 scenario | 1.8683 × 10^6^ | 8.941× 10^5^ | 8.672 × 10^5^ | 1.07× 10^5^ |
| 2050s RCP4.5 scenario | 1.8232 × 10^6^ | 9.218× 10^5^ | 7.991× 10^5^ | 1.023× 10^5^ |
| 2050s RCP8.5 scenario | 1.8988× 10^6^ | 1.0211× 10^6^ | 7.785× 10^5^ | 0.991× 10^5^ |
| 2070s RCP2.6 scenario | 1.8157 × 10^6^ | 8.743× 10^5^ | 8.376× 10^5^ | 1.039× 10^5^ |
| 2070s RCP4.5 scenario | 1.877× 10^6^ | 9.681× 10^5^ | 8.034× 10^5^ | 1.054× 10^5^ |
| 2070s RCP8.5 scenario | 1.9514 × 10^6^ | 1.0302 × 10^6^ | 8.183× 10^5^ | 1.029× 10^5^ |
