# Supplementary material for: Current and future distribution of Forsythia suspensa in China under climate change adopting the MaxEnt model
Source: Front Plant Sci. 2024 Jun 3;15:1394799. doi: 10.3389/fpls.2024.1394799 (PMC11180877; doi:10.3389/fpls.2024.1394799)
Supplement: Supplementary file 1 [file DataSheet_1.zip › Supplementary Material/Supplementary material 8.docx]

Supplementary material 8. The changes of the high-suitability area for *F. suspensa* under different GHG emission scenarios

| GHG emission scenarios | the total area of high-suitability area in 2050s | the total area of high-suitability area in 2070s |
| --- | --- | --- |
| RCP2.6 scenario | 1.07 × 10^5^ km^2^ | 1.039 × 10^5^ km^2^ |
| RCP4.5 scenario | 1.023 × 10^5^ km^2^ | 1.054 × 10^5^ km^2^ |
| RCP8.5 scenario | 9.91× 10^4^ km^2^ | 1.029 × 10^5^ km^2^ |
