# Supplementary figures and images for: Current and future distribution of Forsythia suspensa in China under climate change adopting the MaxEnt model
Source: Front Plant Sci. 2024 Jun 3;15:1394799. doi: 10.3389/fpls.2024.1394799 (PMC11180877; doi:10.3389/fpls.2024.1394799)

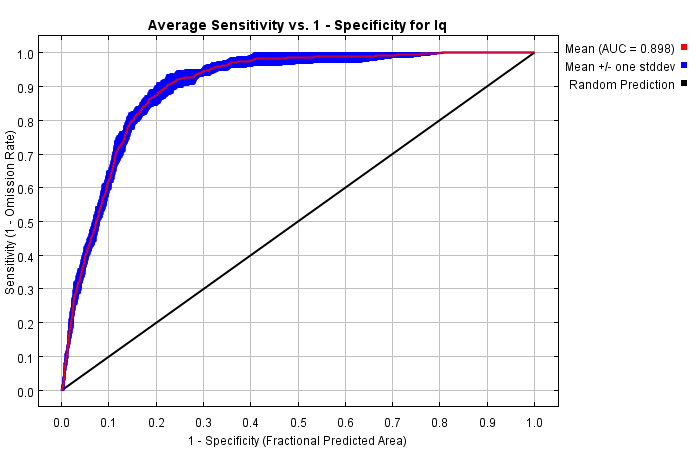


**Supplementary material 2.** The receiver operating characteristic (ROC) curve for *F. suspensa*

Supplement: Supplementary file 1 [file DataSheet_1.zip › Supplementary Material/Supplementary material 2.docx]

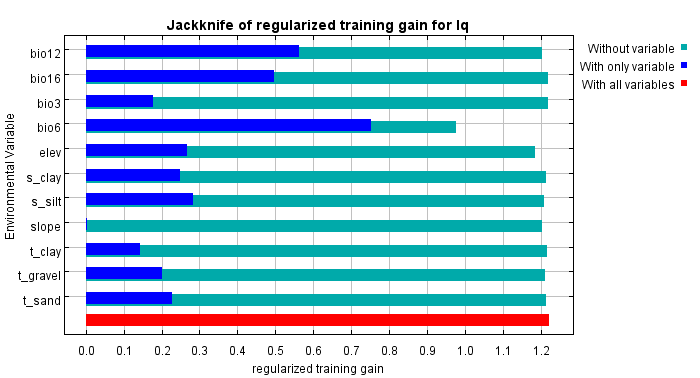


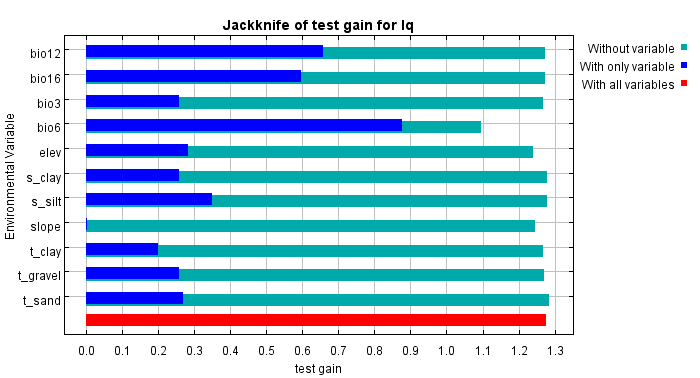


**Supplementary material 3.** The results of the jackknife test of variable importance

Supplement: Supplementary file 1 [file DataSheet_1.zip › Supplementary Material/Supplementary Material 3.docx]

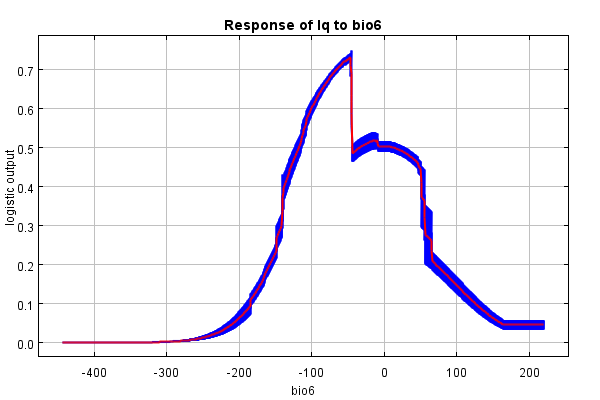

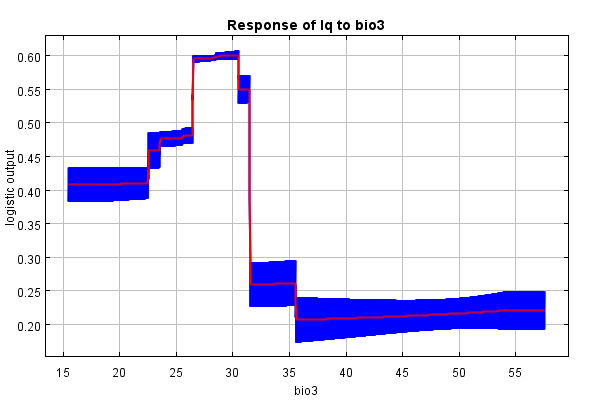


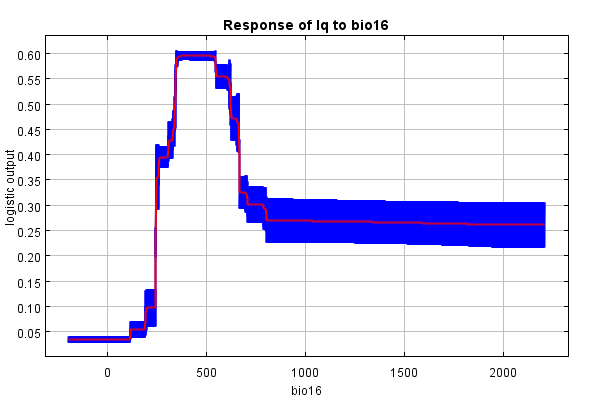

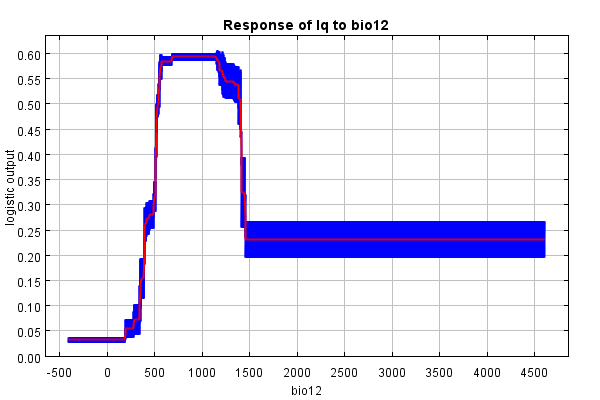


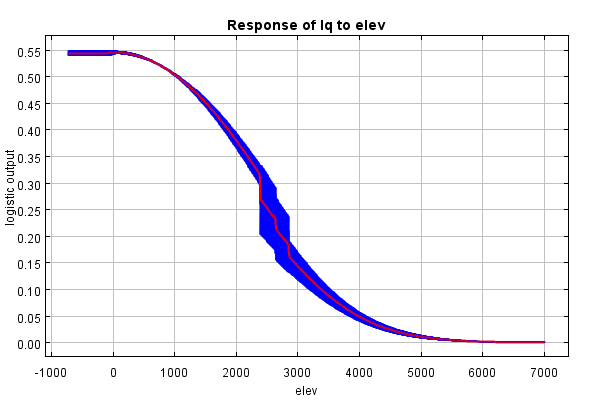

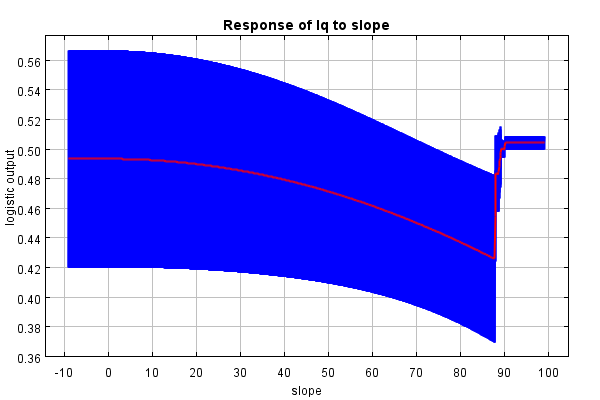


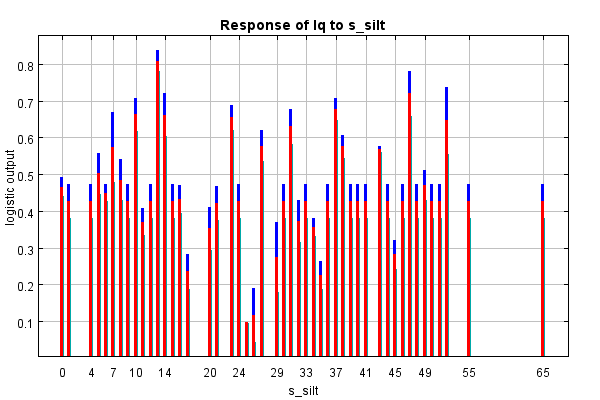

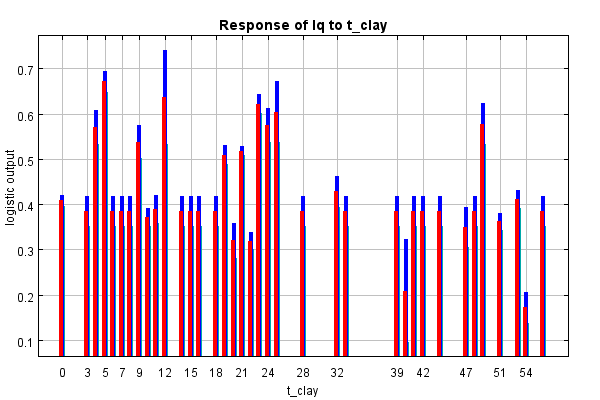


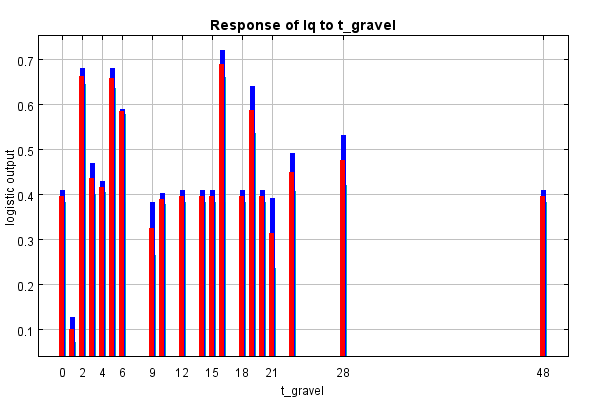

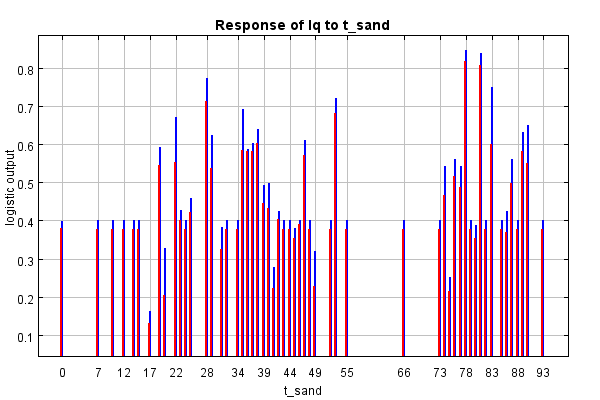


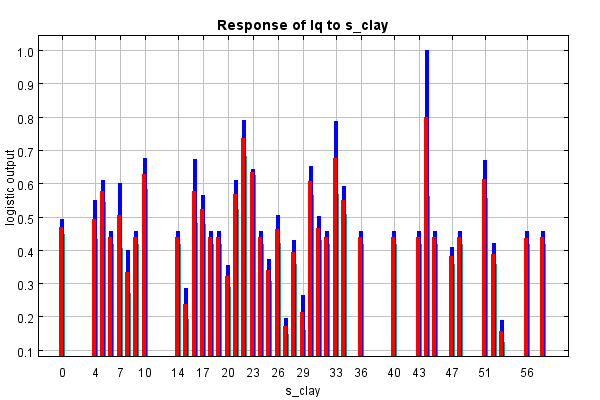


**Supplementary material 4.** Response curves of major environmental variables

Supplement: Supplementary file 1 [file DataSheet_1.zip › Supplementary Material/Supplementary material 4.docx]

Supplementary material 7. Predicted potential distribution of *F. suspensa* in China in the 2050s and 2070s


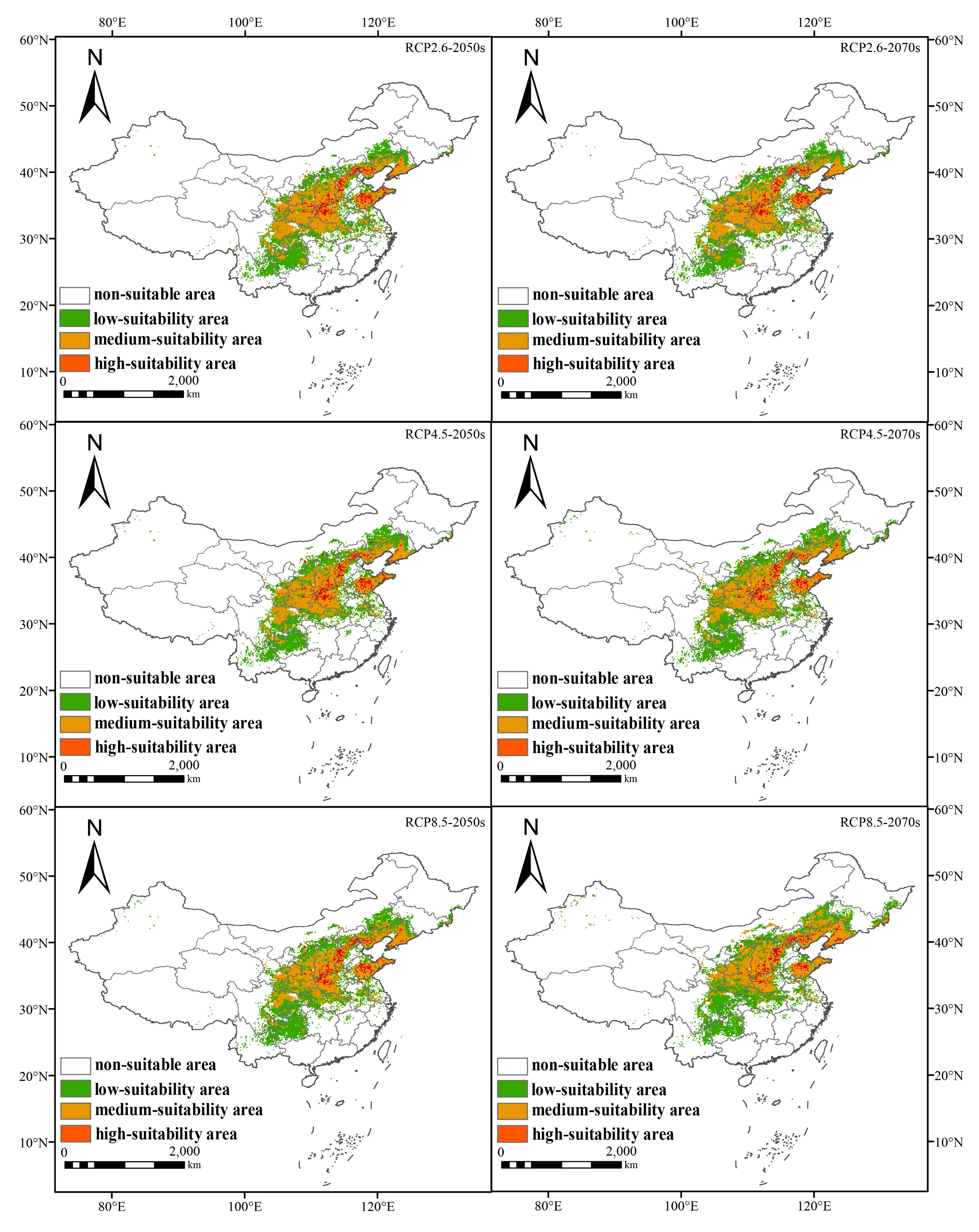

Supplement: Supplementary file 1 [file DataSheet_1.zip › Supplementary Material/Supplementary Material 7.docx]
